# Design of multi-epitope vaccine based on G2c genotype PEDV antigen and its immunogenicity and protective effect in piglets

**DOI:** 10.3389/fvets.2026.1942409

**Published:** 2026-10-08

**Authors:** Hao Luo, Zaixing Sun, Jingxin Wang, Lin Lan, Baoning Wang

**Affiliations:** 1 West China College of Basic Medicine and Forensic Medicine, Sichuan University, Chengdu, China; 2 Department of Biological Medicine, Xihua University, Chengdu, China; 3 Chengdu Textile College, Chengdu, China

**Keywords:** genotype G2c, immunoinformatics, multi-epitope vaccine, neutralizing antibody, PEDV, piglets, protective efficacy

## Abstract

Porcine epidemic diarrhea virus (PEDV) remains a clinically important enteric virus, causing diarrhea, vomiting, dehydration, and high mortality in piglets. As the currently available vaccines may provide inconsistent protection against genetically heterogeneous field strains, particularly G2c variants, improved immunization strategies are still needed. In this study, a multi-epitope vaccine candidate was designed from the PEDV S, M, and N proteins using an immunoinformatics-based workflow. The final construct incorporated 8 linear B-cell epitopes, 1 CTL epitope, and 5 HTL epitopes, and was predicted to contain 6 discontinuous B-cell epitopes; porcine β-defensin-1 (PBD-1) was used as an adjuvant component and attached to the N-terminus. Computational analyses indicated favorable antigenicity, solubility, and structural stability. *In vivo* evaluation showed that the SMN peptide vaccine elicited PEDV-specific IgG, IgA, cytokine, and neutralizing-antibody responses. Following challenge with a virulent G2c PEDV strain, all five piglets in each peptide-vaccinated group survived, compared with three of five piglets in the commercial inactivated-vaccine group and none of the five piglets in the PBS group. These results indicate that the MEV candidate induced immune responses and improved protective outcomes in piglets, supporting its further development as a PEDV vaccine.

## 1 Introduction

PEDV was first described in the late 1970s as the etiological agent of porcine epidemic diarrhea (PED), a rapidly spreading enteric disease in swine (1). Clinically, PED is mainly characterized by severe diarrhea, vomiting, and dehydration, with the highest mortality typically observed in suckling piglets (2). Although pigs at different developmental stages can be infected, neonatal animals usually experience the greatest disease burden, which can translate into substantial losses for affected farms (3, 4). Transplacental transmission has been documented for systemic porcine pathogens such as classical swine fever virus (CSFV) (5–7), porcine circovirus type 2 (PCV2) (8), and porcine reproductive and respiratory syndrome virus (PRRSV) (9), whereas PEDV is an enteric pathogen transmitted predominantly through the fecal-oral route. Consequently, the protection of piglets is particularly important. In recent years, the global re-emergence and rapid diversification of highly virulent G2c variant strains have challenged the protective efficacy of conventional inactivated or live-attenuated vaccines, supporting continued development of new immunization strategies.

PEDV is classified as an enveloped, positive-sense, single-stranded RNA virus within the order Nidovirales, family Coronaviridae, and genus Alphacoronavirus. Its genome is approximately 28 kb and contains coding regions for the replicase polyprotein, spike (S), ORF3 accessory protein, envelope (E), membrane (M), and nucleocapsid (N) proteins, flanked by untranslated regions and a poly(A) tail. Functionally, the S protein is central to receptor binding and neutralizing-antibody induction, whereas the M and N proteins participate in virion assembly and contribute to antigenic and broader immune recognition.

Although experimental studies have identified compounds and natural products with anti-PEDV activity, no licensed antiviral drug is currently available for reliable field control of PEDV. Vaccination therefore remains the main practical measure for prevention. Its effectiveness, however, is challenged by rapid PEDV genetic variation and by insufficient mucosal protection from some existing vaccine formulations. Multi-epitope vaccine design provides a way to select conserved immunogenic regions rationally and to adjust antigen composition in a flexible manner (10, 11). Here, we developed and evaluated a genotype G2c PEDV multi-epitope vaccine candidate containing epitopes from the S, M and N proteins.

## 2 Materials and methods

### 2.1 Sequence collection and phylogenetic analysis

S1 gene sequences representing 50 PEDV strains with diverse geographic origins and virulence backgrounds were collected from NCBI in March 2023. The full genome of PEDV strain CH/HNLYL/2021 (ON960076.1) was also downloaded from NCBI in FASTA format. The reference strains included in this analysis are listed in Supplementary Table S1. The collected S1 sequences were aligned and analyzed phylogenetically in MEGA 11.0. ClustalW was used for S1-sequence alignment, and phylogenetic relationships were inferred with the neighbor-joining method using 1,000 bootstrap replicates.

### 2.2 Immunoinformatics-based epitope screening and MEV construction

Linear B-cell epitope candidates were screened from the PEDV S, M, and N proteins with BepiPred-2.0. This platform uses a random-forest framework trained on antibody–antigen structural epitope data (12). In the present analysis, a cutoff of 0.5 was applied, corresponding to a sensitivity of 0.58564 and specificity of 0.57158. The predicted peptides were then assessed for antigenicity using VaxiJen v2.0 and for sequence conservation using the IEDB-AR v2.22 epitope conservancy tool (13, 14). Only highly antigenic epitopes with complete sequence conservation were retained for downstream analysis.

NetMHCpan 4.1 was used to screen CTL-associated peptides for predicted binding to the selected swine leukocyte antigen class I (SLA-I) allele. Strong and weak binders were defined using percentile-rank thresholds of 0.5 and 2%, respectively (15). Candidate HTL epitopes were initially screened with NetMHCIIpan 4.0 against H-2-IAu, H-2-IEd, and H-2-IEk (15, 16). The five HTL epitopes incorporated into the original MEV construct were selected based on the initial MHC-II prediction together with sequence conservation, antigenicity, and predicted cytokine-inducing potential. To further assess their relevance to swine MHC-II, these five previously selected peptides were subsequently evaluated using MixMHC2pred v2.1 (17) against four SLA-DR heterodimers: SLA-DRA*01:01 paired with SLA-DRB1*01:01, SLA-DRB1*02:01, SLA-DRB1*04:01, or SLA-DRB1*06:01. This SLA-DR analysis served as a retrospective assessment of the porcine MHC-II binding potential of the five HTL epitopes and did not alter the composition of the experimentally evaluated MEV construct. For each peptide–heterodimer pair, the percentile rank (%Rank) and best-scoring nine-residue core were recorded, with lower percentile ranks indicating stronger predicted binding.

To further characterize functional cytokine potential, IL-10Pred (18), IL-4Pred, IL-2Pred, and IFNepitope (19) were used to predict peptides associated with IL-10, IL-4, IL-2, and IFN-γ induction. Predictions were generated using the default settings of each tool.

The MEV sequence was assembled by arranging the selected CTL, HTL, and dominant B-cell epitopes with appropriate peptide linkers. Porcine β-defensin-1 was attached to the N terminus through an EAAAK linker; KK linkers separated adjacent linear B-cell epitopes; the helical spacer HEYGAEALERAG separated the CTL and HTL regions; GPGPG linkers separated adjacent HTL epitopes; and an RVRR linker preceded the C-terminal His tag (20). This arrangement was selected to maintain separation between functional regions and reduce junction-derived epitopes.

### 2.3 In silico characterization of the MEV construct

ANTIGENpro, VaxiJen v2.0, and AllerTOP v2.0 were applied to evaluate the predicted antigenicity and allergenicity of the vaccine construct (13, 21, 22). SOLpro was used to estimate solubility (23). Additional physicochemical parameters, including molecular weight, theoretical isoelectric point, instability index, aliphatic index, grand average of hydropathy, and estimated half-life, were calculated with ExPASy ProtParam.

Secondary-structure features of the final MEV were predicted using PSIPRED 4.0 and SOPMA (24, 25). These analyses were used to estimate the proportions of alpha-helix, extended strand, beta-turn, and random coil within the designed construct. The tertiary structure of the final vaccine sequence was generated using Robetta (26). The initial structural model was refined with GalaxyRefine (27), and model quality before and after refinement was assessed using Ramachandran plots from EBI-PDBSum (28). Potential disulfide engineering sites were further examined with Disulfide by Design 2.0 (29), while ProSA-web (30) and SAVES v6.0 were used to evaluate global and stereochemical model quality.

After structural refinement and validation, conformational B-cell epitopes were mapped on the MEV tertiary model using ElliPro (31). As an experimentally determined porcine TLR3 ectodomain structure was unavailable, molecular docking was performed between the MEV construct and the experimentally determined human TLR3 ectodomain (PDB ID: 2A0Z, chain A; 2.40 Å resolution) using PyDockWEB (32, 33). The docking complex was examined with LigPlot+ to identify hydrogen bonds and hydrophobic contacts at the binding interface. A porcine TLR3 ectodomain model (UniProt F1RZN4, residues 28–696) was generated with SWISS-MODEL using 2A0Z chain A as the structural template. Sequence identity, GMQE, QMEANDisCo, and QMEAN4 Z-score were used to assess similarity and model quality (34, 35). C-ImmSim was used to simulate immune responses induced by the final vaccine construct (36). Two immunization events were modeled 7 days apart at time steps 1 and 21 (1 time step = 8 h), while the remaining simulation parameters were kept at default settings.

To improve the predicted expression of the vaccine construct in *Escherichia coli*, codon optimization was performed using GenScript. The amino acid sequence was back-translated with Gene Infinity (37), and the optimized DNA sequence was evaluated in GenScript for codon adaptation index (CAI) and GC content.

### 2.4 Recombinant expression, purification, and protein validation

The target sequence was synthesized by GenScript Biotech and inserted into pET-30a(+) for recombinant expression. The pET-30a(+)/MEV plasmid was validated by DNA sequencing and transformed into *E. coli* Rosetta2(DE3) cells. Recombinant MEV protein was purified by Ni^2+^ affinity chromatography. Recombinant MEV expression and solubility were assessed by 12% SDS-PAGE, and protein identity was confirmed by Western blotting. ECL chemiluminescence was used for membrane development, and signals were captured with Image Lab v4.0.1.

### 2.5 Piglet vaccination and challenge design

Twenty 5-week-old piglets were obtained from a commercial farm with no known history of PEDV outbreaks or PEDV vaccination. All animals were housed in temperature-controlled isolation rooms and maintained under the same feeding conditions. The experiment was approved by the Ethics Committee of Chengdu University of Traditional Chinese Medicine (approval number: 2024–049) and conducted in accordance with relevant guidelines and regulations. Piglets were randomly allocated to four groups (*n* = 5 per group) for an initial controlled evaluation of vaccine immunogenicity and protective efficacy. Piglets received the first immunization at 5 weeks of age and were challenged at 10 weeks of age. This schedule allowed completion of the two-dose immunization regimen and development of measurable adaptive immune responses before challenge. The post-weaning challenge model was used to assess vaccine efficacy using immunological, clinical, virological, and survival outcomes, with survival considered as one component of the overall efficacy evaluation. Vaccine doses were selected based on previous PEDV subunit-vaccine studies (38), and the experimental design is summarized in Table 1.

**Table 1:** Experimental design.

Group	Treatment	Dpv	Route	Sample collection	Challenge inoculum (dpv)
1	PBS	0, 14	IM	21, 28, 35	PEDV/CH/HN/NK1/2021 (35 dpv)
2	SMN peptide	0, 14	IM	21, 28, 35	PEDV/CH/HN/NK1/2021 (35 dpv)
3	SMN peptide-ISA 71VG	0, 14	IM	21, 28, 35	PEDV/CH/HN/NK1/2021 (35 dpv)
4	Inactivated vaccine	0, 14	IM	21, 28, 35	PEDV/CH/HN/NK1/2021 (35 dpv)

Dpv denotes days post vaccination, and IM denotes intramuscular injection.

Piglets in the control group received 2 mL of sterile PBS by intramuscular injection. Piglets in the vaccine groups received intramuscular administration of 100 μg peptide, 100 μg peptide formulated with ISA 71VG adjuvant (3.7 v/v), or 2 mL of commercial inactivated PEDV vaccine (inactivated genotype G2c strain; pre-inactivation virus titer ≥10^7^ TCID50). The commercial inactivated vaccine was included as a reference vaccine. At 35 dpv, each piglet was orally challenged with 2 × 10^5^ TCID50 of the highly virulent genotype G2c strain PEDV/CH/HN/NK1/2021. Before challenge, rectal swabs were collected from all piglets and tested negative for PEDV by real-time PCR. Clinical signs were monitored for 7 consecutive days after challenge. Fecal consistency was scored as 0, normal; 1, pasty; 2, semiliquid; and 3, liquid (39). Daily fecal samples were collected throughout the observation period for qRT-PCR detection of PEDV RNA. Piglets meeting any humane-endpoint criterion were immediately euthanized. Predefined humane endpoints included severe persistent diarrhea accompanied by marked dehydration, inability to stand or access feed and water, and pronounced loss of responsiveness. Spontaneous death and euthanasia after reaching a predefined PEDV-related humane endpoint were both treated as death events.

### 2.6 Antibody and virus-neutralization assays

Serum samples were obtained at 21, 28, and 35 dpv, and PEDV-specific IgG and IgA levels were determined by indirect ELISA.

PEDV neutralization assays were used to measure NAb titers against CV777, SCSZ-1, and PEDV/CH/HN/NK1. Vero cells were prepared at 2 × 10^5^ cells/well. Serum samples were serially diluted twofold at eight dilution points and tested in triplicate. Each diluted serum sample was mixed with an equal volume of virus suspension containing 2000 TCID50/mL and incubated at 37 °C for 96 h. Cytopathic effects were recorded, and neutralizing titers were calculated using the Reed–Muench method. Neutralizing-antibody titers were expressed as reciprocal 50% neutralization titers (NT50) and are presented without logarithmic transformation.

### 2.7 Cytokine detection

Serum IFN-γ, IL-10, IL-2, and IL-4 concentrations were quantified with antigen-capture ELISA kits according to the manufacturer’s protocol (Bio-Swamp Biotech Corporation, China). Each assay was performed in triplicate. Absorbance was measured at 450 nm, and OD450 values were converted to concentrations (pg/mL) using the standard curves supplied with the kits.

### 2.8 Viral RNA isolation and real-time PCR

Viral RNA was extracted from daily fecal samples with the Virus Genome DNA/RNA Extraction Kit (Dhelix, China) following the manufacturer’s protocol. PEDV N-gene RNA was detected by qRT-PCR as described previously (40). The raw Ct values obtained from qRT-PCR were used for analysis without normalization to a reference gene. Lower Ct values corresponded to higher levels of PEDV RNA.

### 2.9 Statistical methods

Data were analyzed in GraphPad Prism. Results are expressed as mean ± standard deviation (SD). Repeated-measures data, including antibody levels, cytokine concentrations, neutralizing-antibody titers, body-weight changes, and fecal qRT-PCR Ct values, were analyzed using a two-way mixed-effects model followed by Dunnett’s multiple-comparisons test against the PBS group. Diarrhea scores were compared using the Kruskal–Wallis test followed by Dunn’s multiple-comparisons test. Statistical significance was defined as *p < 0.05, **p < 0.01, and ***p < 0.001. Given the group size of five piglets, survival was summarized descriptively as exact counts and percentages, and no inferential between-group survival test was performed. The repeated-measures and nonparametric analyses described above were applied only to their corresponding outcomes.

## 3 Results

### 3.1 Phylogenetic background and MEV epitope design

S1-based phylogenetic analysis (Figure 1) resolved the 50 PEDV reference strains into three major lineages: S-INDEL, G1, and G2. The G1 lineage contained the classical G1a and G1b subgroups, whereas variant strains were distributed among G2a, G2b, and G2c within the G2 lineage. Most strains reported between 2010 and 2023 clustered with G2, consistent with the current predominance of variant PEDV strains. CH/HNLYL/2021 fell within the G2c branch and was closely related to recent Chinese epidemic isolates, supporting its use as a representative circulating field strain.

**Figure 1:**
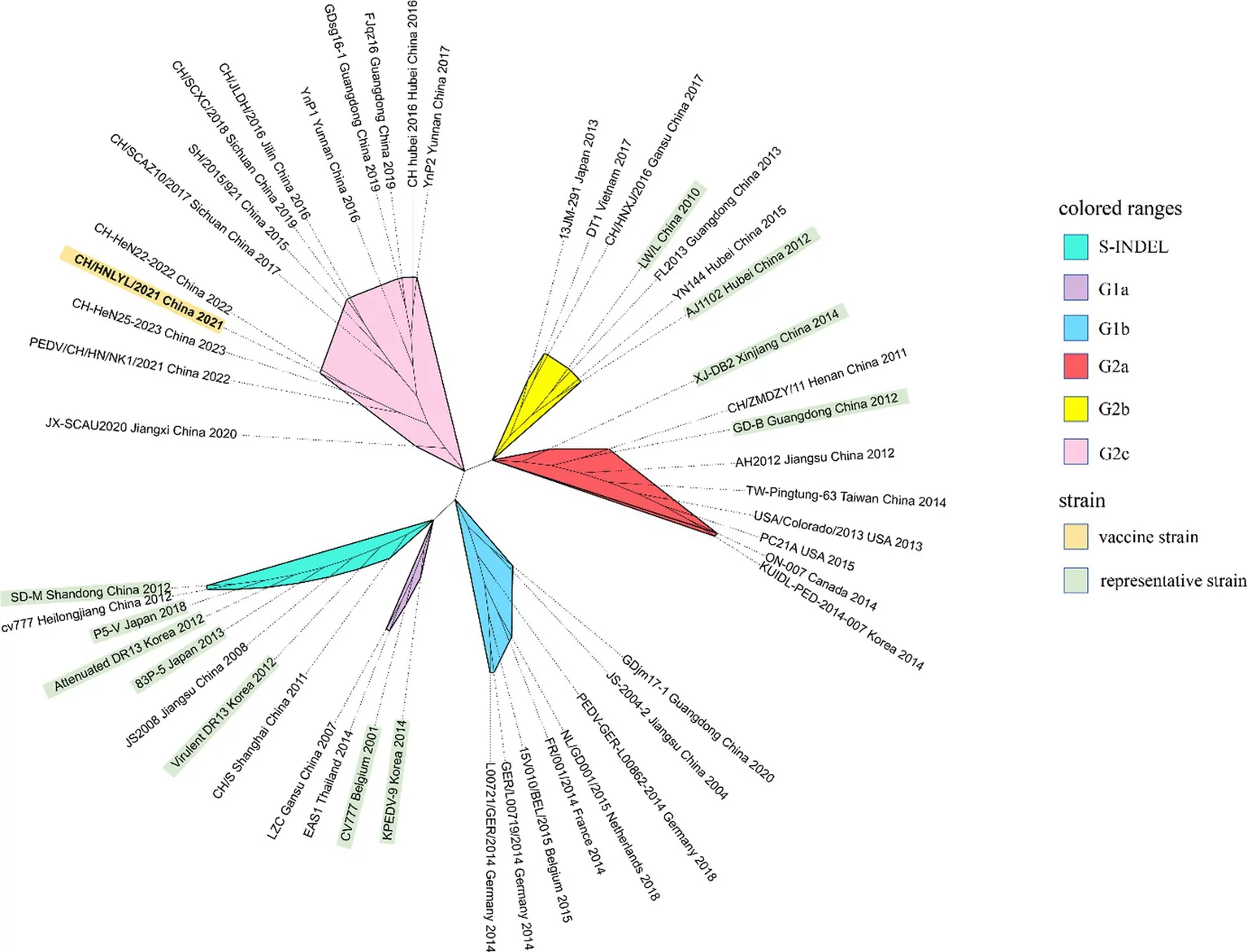
S1-gene-based phylogenetic tree constructed from 50 representative PEDV strains.

BepiPred 2.0 identified eight fully conserved linear B-cell epitopes from the PEDV S, M, and N proteins. The selected CTL epitope AVDGGDTAV was fully conserved, showed predicted binding to SLA-1*04:01, and had a VaxiJen score of 0.4298 (Table 2). Retrospective SLA-DR reassessment of the five HTL epitopes incorporated into the MEV revealed different predicted binding patterns across the tested porcine MHC-II heterodimers (Table 3). TTTIVYGRVGRSVNA showed %Rank ≤ 10 for all four SLA-DR heterodimers, followed by SIGYVPSQSGQVKIA (3/4), RDDLVAAVKDALKS (2/4), and QIRWRMRRGERIEQP (1/4). In contrast, EGVFWVAKEGAKTEP showed %Rank >10 for all four tested heterodimers. Overall, four of the five HTL epitopes showed predicted binding to at least one of the tested SLA-DR heterodimers, whereas EGVFWVAKEGAKTEP did not reach the %Rank ≤ 10 threshold within the four-heterodimer panel evaluated in this study. Predicted IFN-γ, IL-10, IL-2, and IL-4 induction and sequence conservation are summarized in Table 2.

**Table 2:** Summary of selected epitopes incorporated into the MEV construct.

Type	Protein	Allele/MHC context	Position	Epitope	Conservancy	Antigenicity	IFN-γ	IL-10	IL-2	IL-4
Linear B-cell epitopes	S	/	347–354	GYPEFGSG	100%	0.7323	–	–	–	–
	S	/	786–793	TNFSMSIR	100%	2.0025	–	–	–	–
	S	/	842–850	RLESVEVNS	100%	0.8057	–	–	–	–
	M	/	154–165	YKVATGVQVSQL	100%	0.7060	–	–	–	–
	M	/	181–194	GRVGRSVNASSGTG	100%	0.9916	–	–	–	–
	N	/	5–16	SFQDRGRKRVPL	100%	0.9235	–	–	–	–
	N	/	84–91	ADLRYRTR	100%	2.4394	–	–	–	–
	N	/	299–316	GGFKNFGDAEFVEKGVDA	100%	0.8516	–	–	–	–
CTL epitope	N	SLA-1*04:01	422–430	AVDGGDTAV	100%	0.4298	–	–	–	–
HTL epitopes	S	H-2-IAu	761–775	SIGYVPSQSGQVKIA	100%	0.8889	P	P	P	N
	M	H-2-IAu	175–189	TTTIVYGRVGRSVNA	100%	0.8162	N	P	P	P
	N	H-2-IEd	93–107	EGVFWVAKEGAKTEP	100%	0.5174	P	N	P	N
	N	H-2-IEd	56–70	QIRWRMRRGERIEQP	100%	0.8727	N	P	P	P
	N	H-2-IEk	219–232	RDDLVAAVKDALKS	100%	0.5746	N	N	P	N

P, predicted positive; N, predicted negative. H-2 class II alleles indicate the original MHC-II screening context used for HTL epitope selection. The five HTL epitopes were subsequently reassessed against porcine SLA-DR heterodimers, and the detailed results are presented in Table 3.

**Table 3:** Predicted binding of the five selected HTL epitopes to four porcine SLA-DR heterodimers.

HTL epitope	DRB1*01:01	DRB1*02:01	DRB1*04:01	DRB1*06:01	Best SLA-DRB1	Predicted core	Coverage (%Rank ≤ 10)
SIGYVPSQSGQVKIA	26.1	2.36	5.50	0.112	DRB1*06:01	YVPSQSGQV	3/4
TTTIVYGRVGRSVNA	7.06	6.55	0.093	5.15	DRB1*04:01	IVYGRVGRS	4/4
EGVFWVAKEGAKTEP	24.2	20.5	16.1	13.4	DRB1*06:01	WVAKEGAKT	0/4
QIRWRMRRGERIEQP	42.0	12.2	59.9	5.48	DRB1*06:01	WRMRRGERI	1/4
RDDLVAAVKDALKS	10.5	9.22	23.1	3.28	DRB1*06:01	LVAAVKDAL	2/4

SLA-DRA*01:01 was paired with each indicated SLA-DRB1 allele. Values are MixMHC2pred percentile ranks (%Rank), with lower values indicating stronger predicted binding. Coverage indicates the number of tested SLA-DR heterodimers with %Rank ≤ 10. A %Rank ≤ 10 cutoff was used to summarize predicted SLA-DR coverage.

Based on the dominant epitopes identified through immunoinformatics screening, a chimeric MEV was constructed with 8 B-cell epitopes, 1 CTL epitope, and 5 HTL epitopes. PBD-1 was attached to the N terminus through an EAAAK linker. KK linkers separated adjacent B-cell epitopes, HEYGAEALERAG separated the CTL and HTL regions, GPGPG linkers separated adjacent HTL epitopes, and an RVRR linker preceded the C-terminal His tag. The construct organization is shown in Figure 2A.

**Figure 2:**
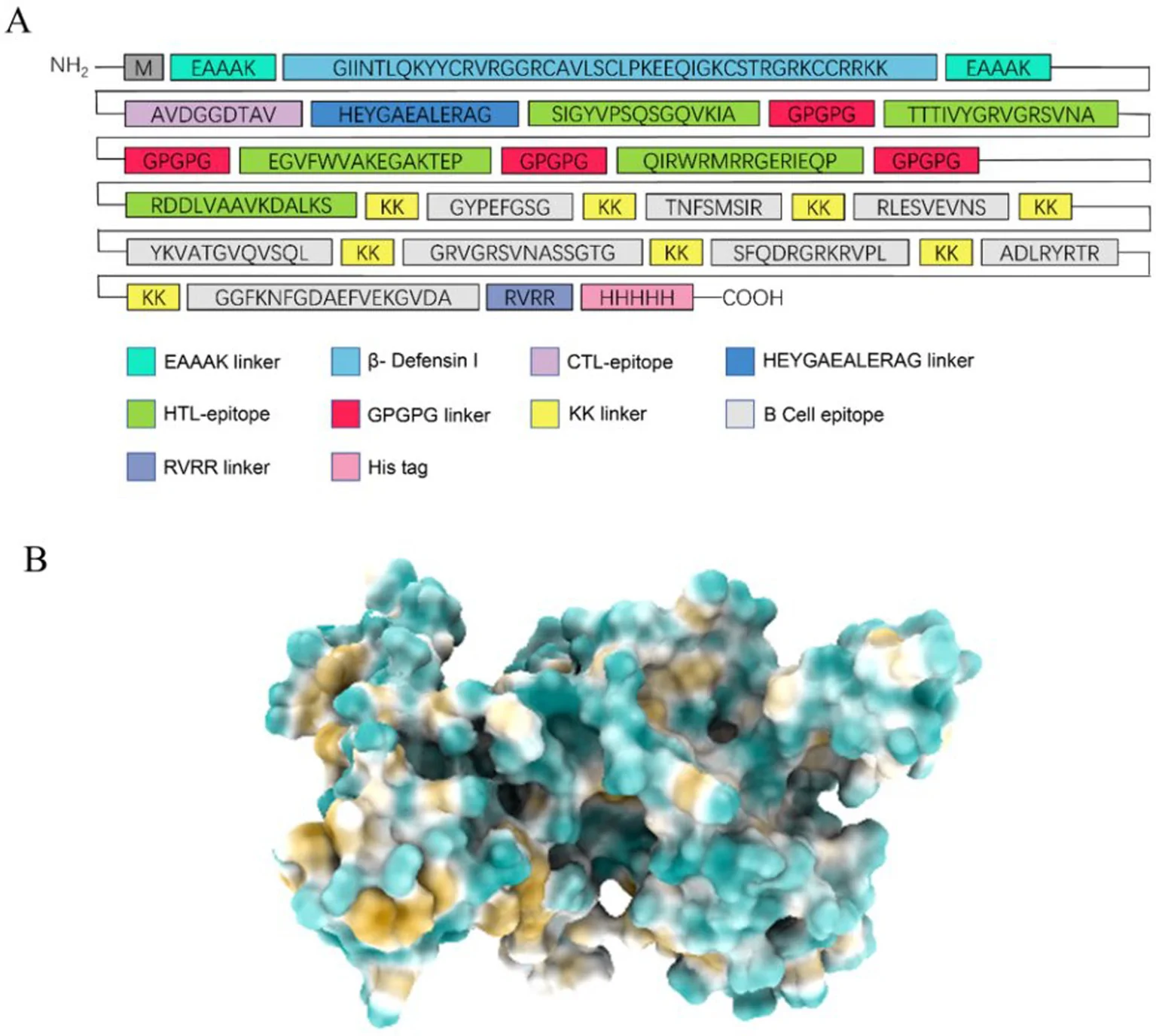
Design and 3D structural modeling of the multi-epitope vaccine (MEV). **(A)** Structural organization of the MEV construct, including the N-terminal PBD-1 adjuvant and the selected linear B-cell, CTL, and HTL epitopes connected by rigid or flexible linkers. **(B)** Robetta-based tertiary model of the MEV protein displayed as the predicted three-dimensional structure.

### 3.2 In silico characterization and structural evaluation of the MEV

ANTIGENpro and VaxiJen v2.0 yielded antigenicity scores of 0.716959 and 0.6860, indicating that the construct had predicted antigenic potential. AllerTOP v2.0 classified the MEV as non-allergenic, and SOLpro predicted a solubility probability of 0.769879, which exceeded the standard cutoff. ProtParam analysis showed that the construct comprised 285 amino acids, with a molecular weight of 30.92 kDa, theoretical pI of 10.36, instability index of 28.96, aliphatic index of 58.84, and GRAVY value of −0.830 (Supplementary Table S2). Together, these parameters support the stability and hydrophilicity of the designed recombinant antigen.

PSIPRED and SOPMA predicted that the MEV contained 23.16% alpha-helix, 24.21% extended strand, 11.23% beta-turn, and 41.40% random coil. Solvent-accessibility analysis indicated that most residues were exposed or moderately exposed, whereas only a small fraction was buried or assigned to disordered regions. This distribution suggests that the construct has favorable antigen accessibility. The optimal MEV tertiary model was generated by Robetta and visualized with UCSF ChimeraX (Figure 2B).

Following GalaxyRefine optimization, the refined model showed improved geometry relative to the initial model (Supplementary Figure S3). The proportion of residues located in the most favored regions of the Ramachandran plot increased from 84.6 to 86.4% after refinement. ProSA-web produced a z-score of −6.37, and the predominantly negative energy profile indicated an acceptable global fold. SAVES v6.0 further supported model quality, with 80.70% of residues showing an average 3D-1D score ≥ 0.1 and an overall quality factor of 87.68. These results indicated that the refined tertiary model was suitable for subsequent epitope mapping and docking analysis. ElliPro identified 6 discontinuous B-cell epitopes on the MEV tertiary model. These epitopes contained 3 to 60 residues and had scores ranging from 0.628 to 0.779 (Supplementary Figure S4; Supplementary Table S3). Because each score exceeded the 0.5 threshold, the construct was predicted to have favorable conformational antigenicity.

Docking of the MEV with the experimentally determined human TLR3 ectodomain generated 101 candidate models. Model 528 ranked first and was selected for interface analysis. Hydrogen bonds were predicted with Glu146, His283, His284, and Arg22, while hydrophobic contacts involved Arg41, His285, Arg43, Lys44, Arg48, Gly20, Met142, Gly42, Gln84, Gly36, and Lys182 (Figure 3). For comparison with the porcine receptor, a porcine TLR3 ectodomain model was generated using human TLR3 (2A0Z) as the template. The porcine sequence shared 83.11% identity with the human template, and the model had a GMQE of 0.649, QMEANDisCo of 0.805 ± 0.053, and QMEAN4 Z-score of −1.008. These results support conservation of the overall TLR3 ectodomain architecture between the two species.

**Figure 3:**
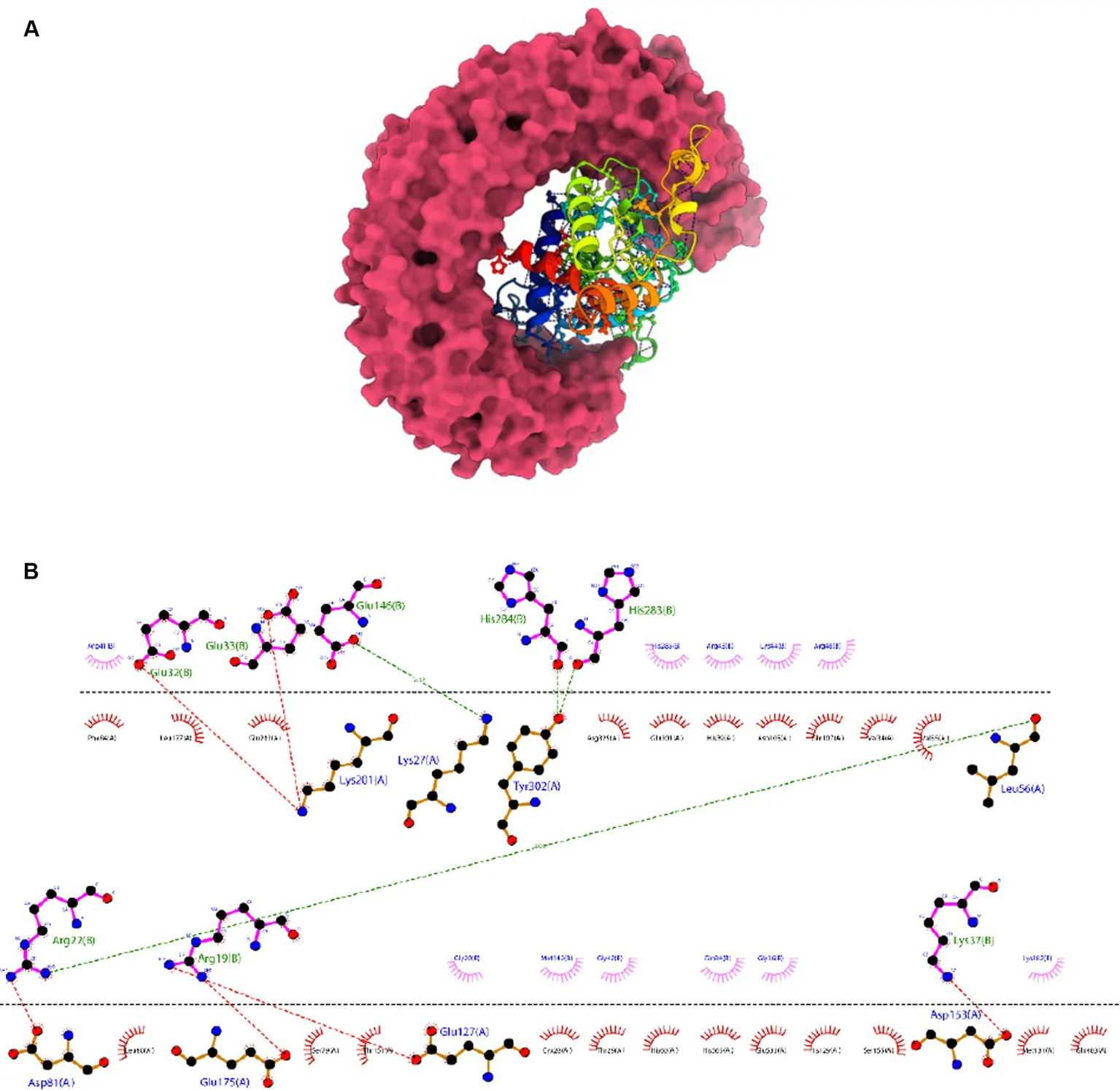
Molecular docking of the MEV with the TLR3 ectodomain. **(A)** Overall docking model of the MEV (ribbon) with the human TLR3 ectodomain (surface; PDB ID: 2A0Z, chain A). **(B)** Two-dimensional interaction map showing predicted hydrogen bonds and hydrophobic contacts at the MEV–TLR3 interface.

### 3.3 Immune-response simulation

C-ImmSim predicted a stronger secondary immune response than the primary response after vaccination. As antigen levels declined, immunoglobulin levels increased, including IgG1 + IgG2, IgM, and combined IgG + IgM. The simulation also indicated sustained B-cell activation, robust helper and cytotoxic T-cell responses, coordinated activation of natural killer cells, dendritic cells, and macrophages, and increased IFN-γ and IL-2. Overall, the predicted immune profile suggests that the MEV can induce both humoral and cellular immunity (Figure 4).

**Figure 4:**
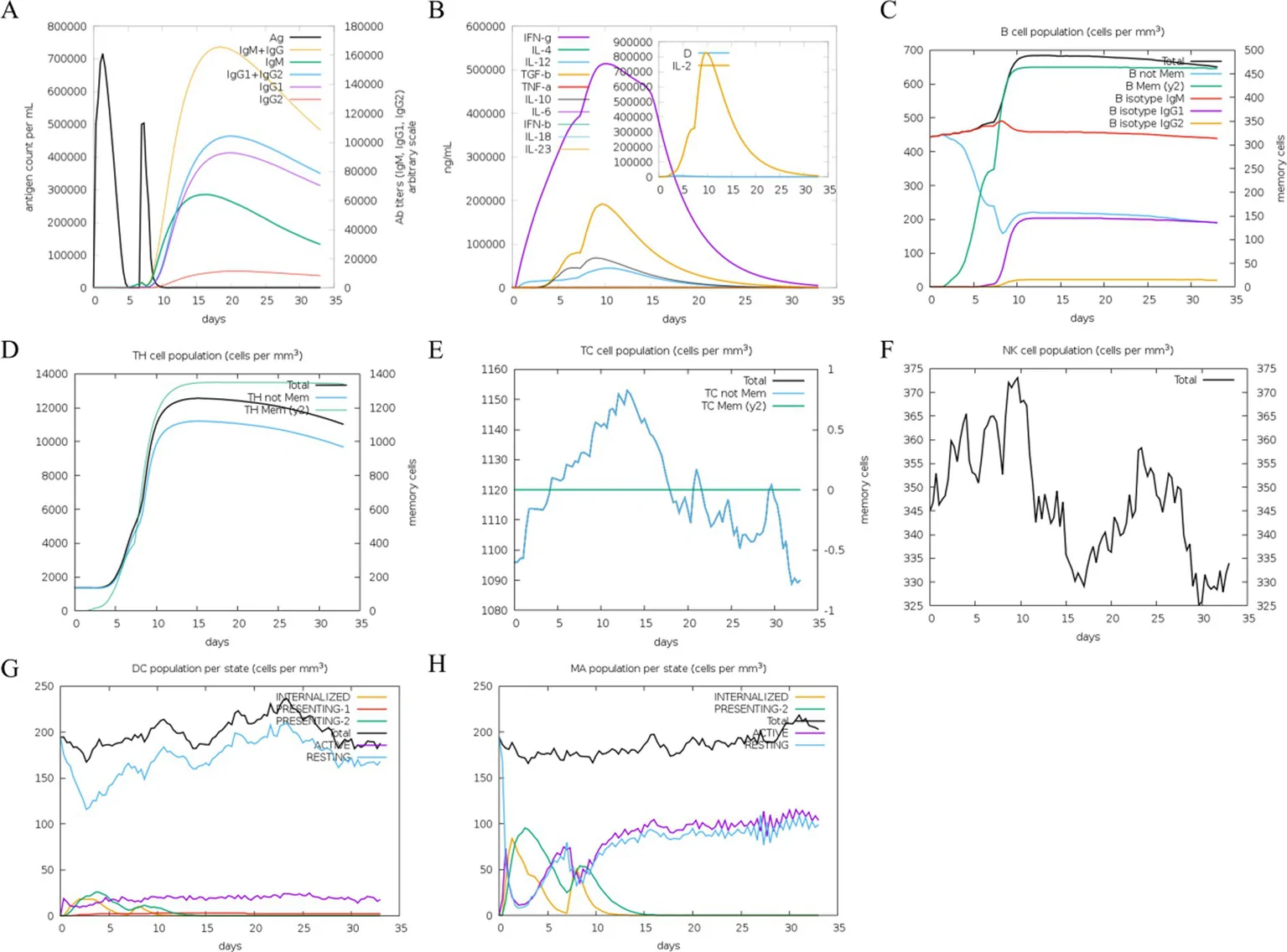
C-ImmSim immune-simulation profile for the final PEDV vaccine candidate. **(A)** Immunoglobulin kinetics after vaccination. **(B)** Cytokine and interleukin profiles. **(C)** B-cell subtype dynamics. **(D)** Memory and non-memory HTL populations. **(E)** Memory and non-memory CTL populations. **(F)** Total NK-cell counts. **(G)** DC populations in different activation states. **(H)** MA-cell populations in different states.

### 3.4 Recombinant expression and validation of the MEV protein

The vaccine construct was reverse-translated into an 855-nucleotide cDNA sequence using Gene Infinity. For *E. coli* expression, GenScript predicted a CAI value of 1.00 and GC content of 61.78%, indicating favorable codon usage and nucleotide composition for the selected host.

GenScript Biotech synthesized the target sequence and inserted it into pET-30a(+) to generate pET-30a(+)/MEV, which was verified by DNA sequencing (Figure 5A). The recombinant plasmid was introduced into *E. coli* Rosetta2(DE3) cells, and the expressed rotein was purified using Ni^2+^ affinity chromatography.

**Figure 5:**
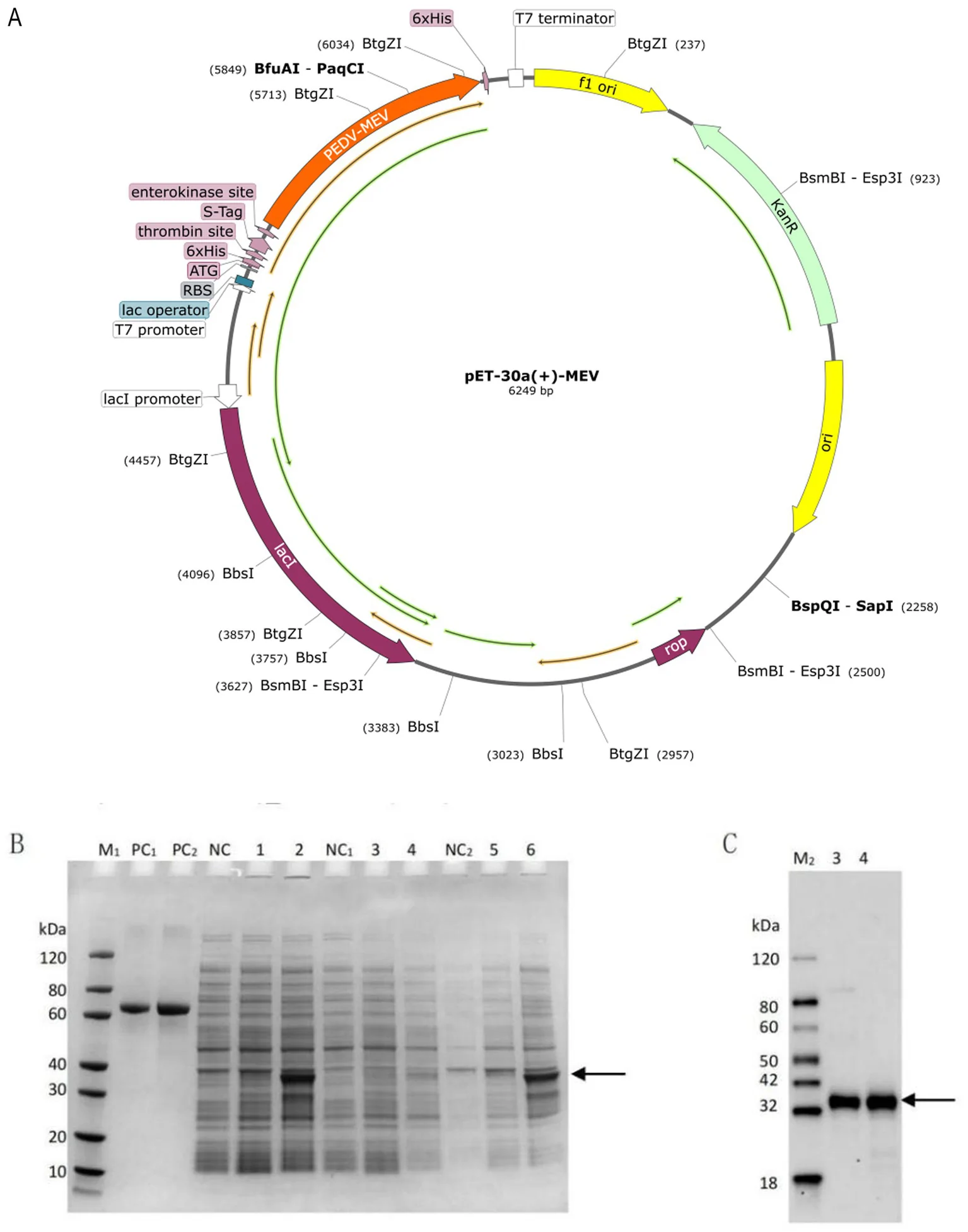
Recombinant protein expression and solubility analysis. **(A)** Prokaryotic expression-vector map of pET-30a(+), showing the cloning sites and optimized reading frame for MEV expression. **(B)** SDS-PAGE analysis: M1, molecular-weight marker; PC1 and PC2, 1 μg and 2 μg BSA controls; NC, uninduced whole-cell lysate; lanes 1 and 2, whole-cell lysates after induction for 16 h at 15 °C and 4 h at 37 °C, respectively; NC1, soluble supernatant from the uninduced culture; lanes 3 and 4, soluble supernatants after the two induction conditions; NC2, pellet from the uninduced culture; lanes 5 and 6, pellets after the two induction conditions. **(C)** Western blotting with an anti-His antibody: M2, molecular-weight marker; lanes 3 and 4, soluble supernatants after induction for 16 h at 15 °C and 4 h at 37 °C, respectively. The arrowed band at approximately 32 kDa corresponds to recombinant MEV.

Recombinant MEV expression and solubility were evaluated by SDS-PAGE (Figure 5B), and protein identity was confirmed by Western blotting (Figure 5C). SDS-PAGE showed a clear band at approximately 32 kDa, consistent with the expected size of the construct. Western blotting with an anti-His monoclonal antibody confirmed the recombinant protein, and predominant detection in the soluble fraction indicated successful soluble expression in *E. coli*.

### 3.5 Antibody responses

Serum IgG and IgA were measured by ELISA to evaluate humoral immunogenicity. At 21, 28, and 35 dpv, all vaccinated groups showed markedly higher PEDV-specific IgG levels than the PBS group (Figure 6A). The SMN peptide–ISA 71VG and commercial inactivated-vaccine groups showed relatively high IgG responses, while the non-adjuvanted SMN peptide group also displayed a sustained increase over time.

**Figure 6:**
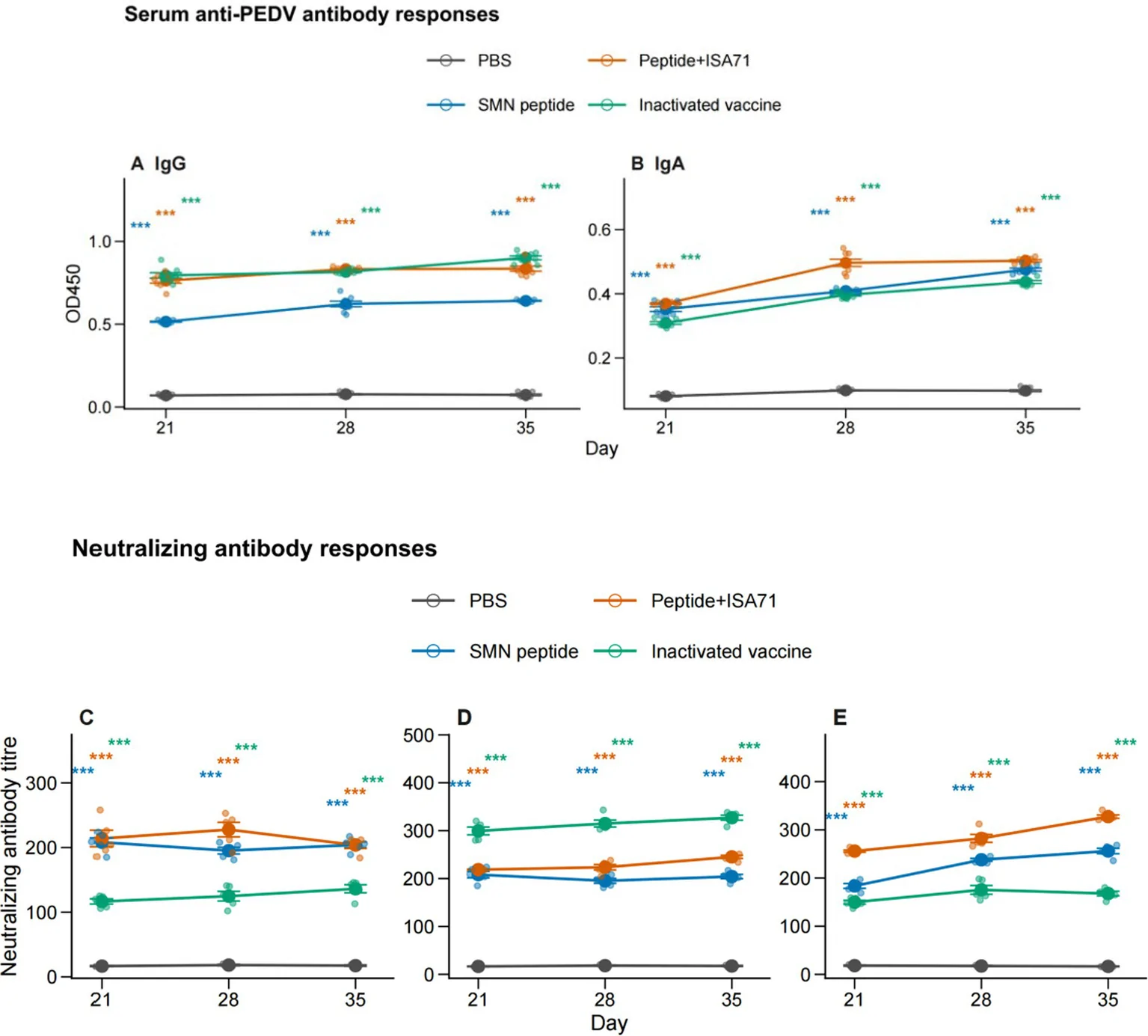
Humoral immune responses and neutralizing activity against three PEDV isolates in piglets. **(A)** Serum PEDV-specific IgG levels measured by ELISA at 21, 28, and 35 dpv. **(B)** Serum PEDV-specific IgA levels measured at the same time points. **(C–E)** Reciprocal 50% neutralization titers (NT50), calculated by the Reed–Muench method, against CV777, SCSZ-1, and CH-HN-NK1. Values are presented as mean ± standard deviation (SD). Significant differences versus the PBS group are indicated by asterisks (*p < 0.05, **p < 0.01, ***p < 0.001).

Serum IgA followed a comparable trend. Relative to PBS controls, piglets receiving SMN peptide, SMN peptide–ISA 71VG, or the commercial inactivated vaccine had clearly increased serum IgA levels from 21 to 35 dpv (Figure 6B). The SMN peptide–ISA 71VG group showed numerically higher serum IgA levels at several time points, consistent with an enhanced humoral response after adjuvant formulation.

### 3.6 Virus-neutralization analysis

The neutralizing activity elicited by the SMN peptide vaccine was assessed against three PEDV strains: CV777, SCSZ-1, and CH-HN-NK1.

Virus neutralization analysis detected reciprocal NT50 responses in all vaccinated groups, whereas the PBS group remained negative (Figures 6C–E). Against the G1a strain CV777, both peptide-immunized groups showed higher numerical titers than the commercial inactivated-vaccine group. For the homologous G2c strain CH-HN-NK1, the SMN peptide–ISA 71VG group showed the highest numerical response, followed by the non-adjuvanted SMN peptide group. In contrast, the commercial inactivated vaccine showed the highest numerical titers against the G2b strain SCSZ-1. Overall, the peptide vaccine induced neutralizing activity against all three tested PEDV isolates.

### 3.7 Cytokine-response analysis

To characterize systemic cytokine responses, serum IL-2, IL-4, IL-10, and IFN-γ were measured by ELISA. Cytokine concentrations were elevated in all vaccinated groups compared with the PBS group across the tested time points (Figure 7). The simultaneous increases in IFN-γ and IL-2, together with IL-4 and IL-10, indicate that vaccination induced a mixed Th1- and Th2-related cytokine response. The SMN peptide–ISA 71VG group showed numerically higher concentrations for most measured cytokines.

**Figure 7:**
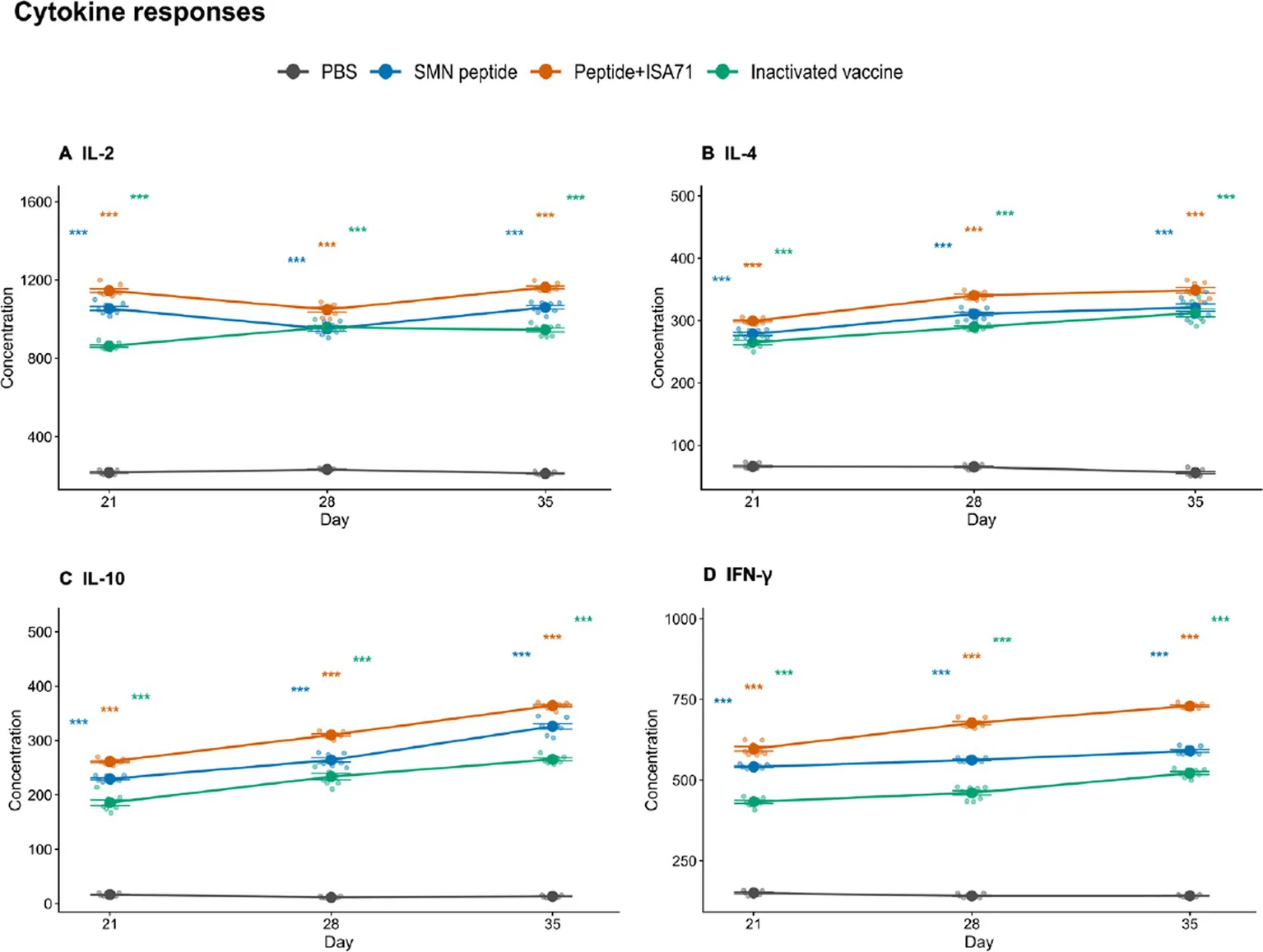
Systemic serum cytokine responses post-vaccination. Peripheral-blood cytokine levels (IL-2, IL-4, IL-10, and IFN-γ) were measured by ELISA to characterize systemic cytokine responses. **(A–D)** Serum concentrations of IL-2, IL-4, IL-10, and IFN-γ at 21, 28, and 35 dpv. Data are shown as mean ± SD (*n* = 5 per group). Significant differences versus the PBS group are marked with asterisks (*p < 0.05, **p < 0.01, ***p < 0.001).

### 3.8 Clinical outcomes after PEDV challenge

Protective efficacy was evaluated by orally challenging the four groups at 35 dpv with 2 × 10^5^ TCID50 of the highly virulent genotype G2c PEDV strain PEDV/CH/HN/NK1/2021 (Figure 8). Before challenge, all piglets were clinically healthy and tested negative for PEDV by real-time PCR. After challenge, the PBS group developed disease earlier and more severely than the vaccinated groups, whereas disease onset was delayed in the SMN peptide and SMN peptide–ISA 71VG groups. During the 7-day observation period, all piglets survived in the SMN peptide group (5/5) and the SMN peptide–ISA 71VG group (5/5), compared with three of five piglets in the commercial inactivated-vaccine group and none of the five piglets in the PBS group. The SMN peptide–ISA 71VG group showed relatively stable daily weight gain, while PBS controls exhibited the greatest weight loss. Fecal Ct values varied during the observation period. Compared with the PBS group, vaccinated groups showed higher Ct values at several time points, corresponding to lower levels of detectable PEDV RNA during these periods. Together, the clinical, survival, and virological findings show that both peptide formulations improved outcomes following G2c PEDV challenge.

**Figure 8:**
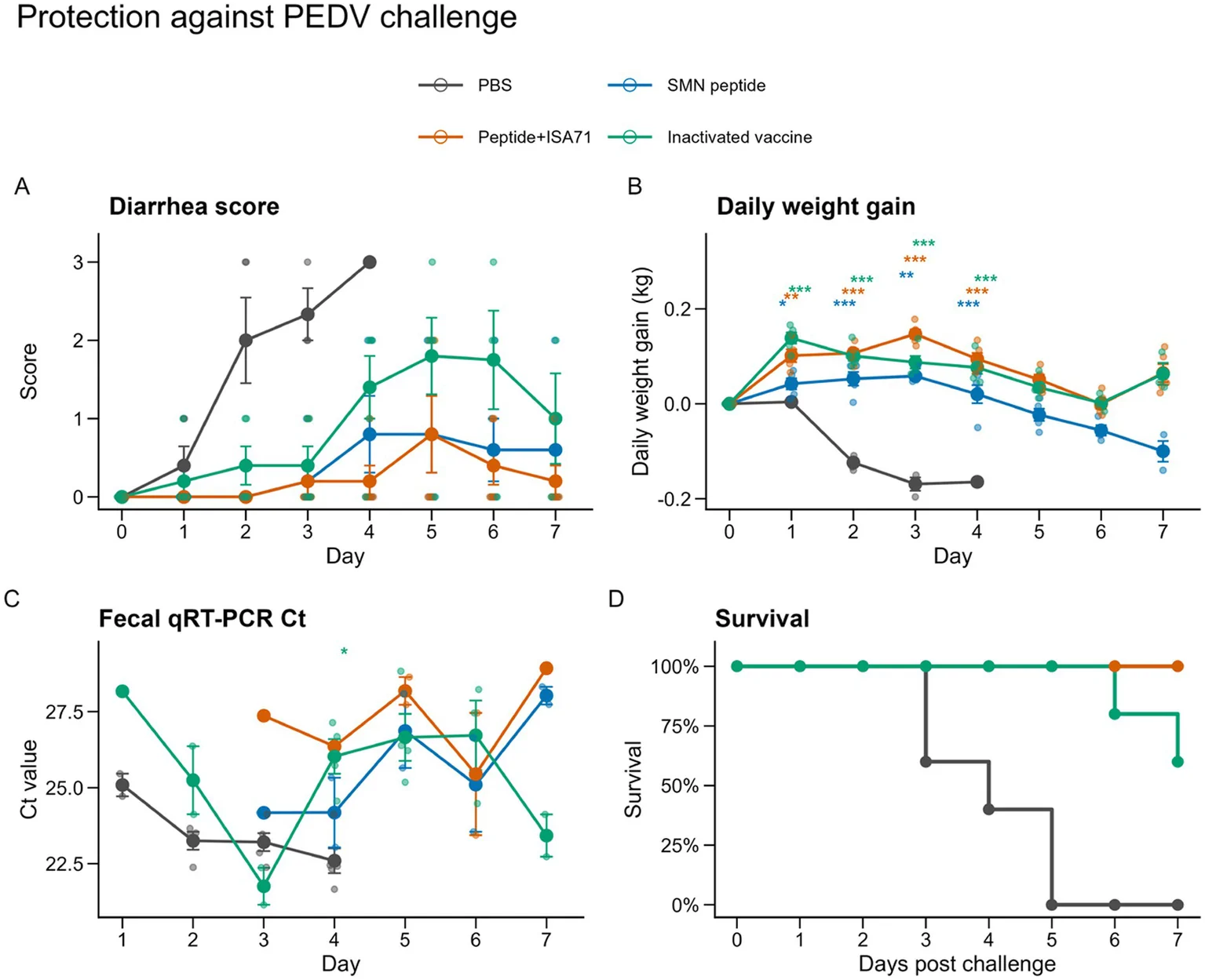
Clinical outcomes after G2c PEDV challenge. **(A)** Daily diarrhea scores recorded for 7 days post-challenge (dpc). **(B)** Average daily weight gain (ADG) during the challenge period. **(C)** Fecal PEDV RNA detected by qRT-PCR and expressed as raw Ct values; lower Ct values indicate higher PEDV RNA levels. **(D)** Survival after challenge: SMN peptide, 5/5; SMN peptide + ISA 71VG, 5/5; commercial inactivated vaccine, 3/5; and PBS, 0/5. Significant differences versus the PBS group are indicated by asterisks for the analyzed non-survival outcomes (*p < 0.05, **p < 0.01, ***p < 0.001).

## 4 Discussion

This study combined immunoinformatics-guided antigen design with piglet-based validation to develop a multi-epitope vaccine candidate targeting a PEDV G2c strain. The construct incorporated conserved epitopes from the S, M, and N proteins, together with N-terminal PBD-1 as an immunostimulatory component. The inclusion of epitopes from multiple structural proteins was intended to broaden antigenic coverage and combine humoral and T-cell-related targets within a single recombinant antigen. The final construct showed favorable predicted antigenicity, solubility, and structural properties, supporting its further experimental evaluation.

The retrospective SLA-DR analysis provided additional information on the predicted porcine MHC-II relevance of the five HTL epitopes incorporated into the construct. TTTIVYGRVGRSVNA showed predicted binding across all four tested SLA-DR heterodimers, while SIGYVPSQSGQVKIA, RDDLVAAVKDALKS, and QIRWRMRRGERIEQP showed allele-dependent binding patterns. In contrast, EGVFWVAKEGAKTEP did not meet the %Rank ≤ 10 criterion for any of the four tested heterodimers. The CTL epitope AVDGGDTAV was selected by considering its complete sequence conservation and predicted binding to SLA-1*04:01 together with its VaxiJen score of 0.4298. These results support considering MHC binding, sequence conservation, and antigenicity together during epitope evaluation. SLA-based prediction tools such as PigMatrix provide complementary approaches for assessing swine T-cell epitopes (41). Because of SLA diversity and the limited four-heterodimer panel examined here, and because the experimental piglets were not SLA genotyped, broader SLA-II coverage and experimental peptide–SLA-II binding should be evaluated in future studies (42).

Because an experimentally determined porcine TLR3 ectodomain structure was unavailable, the human TLR3 ectodomain structure (PDB ID: 2A0Z) was used as a structural reference and as the template for construction of a porcine TLR3 homology model. Docking with 2A0Z suggested a potential MEV–TLR3 interaction surface. The 83.11% human–porcine sequence identity and the overall similarity of the porcine homology model provided structural support for this analysis. However, species-specific differences at the local binding interface may affect the predicted interaction. Therefore, binding to and activation of porcine TLR3 require further experimental validation.

Vaccination induced PEDV-specific serum IgG and IgA, increased serum cytokine concentrations, and elicited neutralizing activity against all three PEDV isolates tested. The simultaneous increases in IFN-γ and IL-2 together with IL-4 and IL-10 indicate a mixed Th1- and Th2-related systemic cytokine response. These findings support the systemic immunogenicity of the MEV formulations. Because IgA was measured in serum, however, the present results do not directly demonstrate intestinal mucosal immunity. Further studies examining intestinal IgA, other mucosal responses, and antigen-specific T-cell responses would help clarify the mechanisms contributing to protection against PEDV infection.

The challenge was performed at 10 weeks of age to allow completion of the two-dose immunization regimen and development of measurable adaptive immunity before challenge. Thus, this post-weaning model was intended to compare vaccine-associated immunological, clinical, virological, and survival outcomes rather than to reproduce the high mortality observed in neonatal PEDV infection. The study included five piglets per group and used a single G2c challenge strain; therefore, the magnitude and reproducibility of protection should be confirmed in larger studies. Further work should also examine long-term immunity, optimal dosing, mucosal responses, and protection against genetically diverse PEDV field strains. Overall, the present findings support further development of the multi-epitope vaccine strategy for PEDV.

## Data Availability

The datasets presented in this study can be found in online repositories. The names of the repository/repositories and accession number(s) can be found in the article/Supplementary material.

## References

[1] Wood EN. An apparently new syndrome of porcine epidemic diarrhoea. Vet Rec. (1977) 100:243–4. doi: 10.1136/vr.100.12.243, 888300

[2] Song D Moon H Kang B. Porcine epidemic diarrhea: a review of current epidemiology and available vaccines. Clin Exp Vaccine Res. (2015) 4:166–76. doi: 10.7774/cevr.2015.4.2.166, 26273575 PMC4524901

[3] Alvarez J Sarradell J Morrison R Perez A. Impact of porcine epidemic diarrhea on performance of growing pigs. PLoS One. (2015) 10:e0120532. doi: 10.1371/journal.pone.0120532, 25768287 PMC4359118

[4] Annamalai T Saif LJ Lu Z Jung K. Age-dependent variation in innate immune responses to porcine epidemic diarrhea virus infection in suckling versus weaned pigs. Vet Immunol Immunopathol. (2015) 168:193–202. doi: 10.1016/j.vetimm.2015.09.006, 26433606 PMC7112776

[5] Park Y Oh Y Wang M Ganges L Bohórquez JA Park S . A novel E2 glycoprotein subunit marker vaccine produced in plant is able to prevent classical swine fever virus vertical transmission after double vaccination. Vaccines (Basel). (2021) 9:418. doi: 10.3390/vaccines9050418, 33922120 PMC8143534

[6] Dewulf J Laevens H Koenen F Mintiens K de Kruif A. An experimental infection with classical swine fever virus in pregnant sows: transmission of the virus, course of the disease, antibody response and effect on gestation. J Vet Med B Infect Dis Vet Public Health. (2001) 48:583–91. doi: 10.1046/j.1439-0450.2001.00467.x 11708677

[7] Henke J Carlson J Zani L Leidenberger S Schwaiger T Schlottau K . Protection against transplacental transmission of moderately virulent classical swine fever virus using live marker vaccine "CP7_E2alf". Vaccine. (2018) 36:4181–7. doi: 10.1016/j.vaccine.2018.06.014, 29895502

[8] Park JS Kim J Ha Y Jung K Choi C Lim JK . Birth abnormalities in pregnant sows infected intranasally with porcine circovirus 2. J Comp Pathol. (2005) 132:139–44. doi: 10.1016/j.jcpa.2004.09.003, 15737340

[9] Martín-Valls GE Hidalgo M Cano E Mateu E. Testing of umbilical cords by real time PCR is suitable for assessing vertical transmission of porcine reproductive and respiratory syndrome virus under field conditions. Vet J. (2018) 234:27–9. doi: 10.1016/j.tvjl.2018.01.008, 29680389

[10] Imdhiyas M Sen S Barman N Buragohain L Malik Y Kumar S. Computational analysis of immunogenic epitopes in the p30 and p54 proteins of African swine fever virus. J Biomol Struct Dyn. (2023) 41:7480–9. doi: 10.1080/07391102.2022.2123400, 36148815

[11] Li G Shu J Jin J Shu J Feng H Chen J . Development of a multi-epitope vaccine for mycoplasma hyopneumoniae and evaluation of its immune responses in mice and piglets. Int J Mol Sci. (2022) 23:7899. doi: 10.3390/ijms23147899, 35887246 PMC9318870

[12] Jespersen MC Peters B Nielsen M Marcatili P. BepiPred-2.0: improving sequence-based B-cell epitope prediction using conformational epitopes. Nucleic Acids Res. (2017) 45:W24–9. doi: 10.1093/nar/gkx346, 28472356 PMC5570230

[13] Doytchinova IA Flower DR. VaxiJen: a server for prediction of protective antigens, tumour antigens and subunit vaccines. BMC Bioinformatics. (2007) 8:4. doi: 10.1186/1471-2105-8-4, 17207271 PMC1780059

[14] Dhanda SK Mahajan S Paul S Yan Z Kim H Jespersen MC . IEDB-AR: immune epitope database-analysis resource in 2019. Nucleic Acids Res. (2019) 47:W502–6. doi: 10.1093/nar/gkz452, 31114900 PMC6602498

[15] Reynisson B Alvarez B Paul S Peters B Nielsen M. NetMHCpan-4.1 and NetMHCIIpan-4.0: improved predictions of MHC antigen presentation by concurrent motif deconvolution and integration of MS MHC eluted ligand data. Nucleic Acids Res. (2020) 48:W449–54. doi: 10.1093/nar/gkaa379, 32406916 PMC7319546

[16] Reynisson B Barra C Kaabinejadian S Hildebrand WH Peters B Nielsen M. Improved prediction of MHC II antigen presentation through integration and motif deconvolution of mass spectrometry MHC eluted ligand data. J Proteome Res. (2020) 19:2304–15. doi: 10.1021/acs.jproteome.9b00874, 32308001

[17] Racle J Guillaume P Schmidt J Michaux J Larabi A Lau K . Machine learning predictions of MHC-II specificities reveal alternative binding mode of class II epitopes. Immunity. (2023) 56:1359–1375.e13. doi: 10.1016/j.immuni.2023.03.009, 37023751

[18] Nagpal G Usmani SS Dhanda SK Kaur H Singh S Sharma M . Computer-aided designing of immunosuppressive peptides based on IL-10 inducing potential. Sci Rep. (2017) 7:42851. doi: 10.1038/srep42851, 28211521 PMC5314457

[19] Dhanda SK Vir P Raghava GP. Designing of interferon-gamma inducing MHC class-II binders. Biol Direct. (2013) 8:30. doi: 10.1186/1745-6150-8-30, 24304645 PMC4235049

[20] Tarrahimofrad H Rahimnahal S Zamani J Jahangirian E Aminzadeh S. Designing a multi-epitope vaccine to provoke the robust immune response against influenza a H7N9. Sci Rep. (2021) 11:24485. doi: 10.1038/s41598-021-03932-2, 34966175 PMC8716528

[21] Magnan CN Zeller M Kayala MA Vigil A Randall A Felgner PL . High-throughput prediction of protein antigenicity using protein microarray data. Bioinformatics. (2010) 26:2936–43. doi: 10.1093/bioinformatics/btq551, 20934990 PMC2982151

[22] Dimitrov I Bangov I Flower DR Doytchinova I. AllerTOP v.2--a server for in silico prediction of allergens. J Mol Model. (2014) 20:2278. doi: 10.1007/s00894-014-2278-5 24878803

[23] Magnan CN Randall A Baldi P. SOLpro: accurate sequence-based prediction of protein solubility. Bioinformatics. (2009) 25:2200–7. doi: 10.1093/bioinformatics/btp386, 19549632

[24] Buchan DW Minneci F Nugent TC Bryson K Jones DT. Scalable web services for the PSIPRED protein analysis workbench. Nucleic Acids Res. (2013) 41:W349–57. doi: 10.1093/nar/gkt381, 23748958 PMC3692098

[25] Geourjon C Deléage G. SOPMA: significant improvements in protein secondary structure prediction by consensus prediction from multiple alignments. Comput Appl Biosci. (1995) 11:681–4. doi: 10.1093/bioinformatics/11.6.681, 8808585

[26] Kim DE Chivian D Baker D. Protein structure prediction and analysis using the Robetta server. Nucleic Acids Res. (2004) 32:W526–31. doi: 10.1093/nar/gkh468, 15215442 PMC441606

[27] Heo L Park H Seok C. GalaxyRefine: protein structure refinement driven by side-chain repacking. Nucleic Acids Res. (2013) 41:W384–8. doi: 10.1093/nar/gkt458, 23737448 PMC3692086

[28] Laskowski RA. PDBsum: summaries and analyses of PDB structures. Nucleic Acids Res. (2001) 29:221–2. doi: 10.1093/nar/29.1.221, 11125097 PMC29784

[29] Craig DB Dombkowski AA. Disulfide by design 2.0: a web-based tool for disulfide engineering in proteins. BMC Bioinformatics. (2013) 14:346. doi: 10.1186/1471-2105-14-346, 24289175 PMC3898251

[30] Wiederstein M Sippl MJ. ProSA-web: interactive web service for the recognition of errors in three-dimensional structures of proteins. Nucleic Acids Res. (2007) 35:W407–10. doi: 10.1093/nar/gkm290, 17517781 PMC1933241

[31] Ponomarenko J Bui HH Li W Fusseder N Bourne PE Sette A . ElliPro: a new structure-based tool for the prediction of antibody epitopes. BMC Bioinformatics. (2008) 9:514. doi: 10.1186/1471-2105-9-514, 19055730 PMC2607291

[32] Jiménez-García B Pons C Fernández-Recio J. pyDockWEB: a web server for rigid-body protein-protein docking using electrostatics and desolvation scoring. Bioinformatics. (2013) 29:1698–9. doi: 10.1093/bioinformatics/btt262, 23661696

[33] Bell JK Botos I Hall PR Askins J Shiloach J Segal DM . The molecular structure of the toll-like receptor 3 ligand-binding domain. Proc Natl Acad Sci USA. (2005) 102:10976–80. doi: 10.1073/pnas.0505077102, 16043704 PMC1182468

[34] Waterhouse A Bertoni M Bienert S Studer G Tauriello G Gumienny R . SWISS-MODEL: homology modelling of protein structures and complexes. Nucleic Acids Res. (2018) 46:W296–303. doi: 10.1093/nar/gky427, 29788355 PMC6030848

[35] Studer G Rempfer C Waterhouse AM Gumienny R Haas J Schwede T. QMEANDisCo—distance constraints applied on model quality estimation. Bioinformatics. (2020) 36:1765–71. doi: 10.1093/bioinformatics/btz828, 31697312 PMC7075525

[36] Rapin N Lund O Bernaschi M Castiglione F. Computational immunology meets bioinformatics: the use of prediction tools for molecular binding in the simulation of the immune system. PLoS One. (2010) 5:e9862. doi: 10.1371/journal.pone.0009862, 20419125 PMC2855701

[37] Stothard P. The Sequence Manipulation Suite: JavaScript programs for analyzing and formatting protein and DNA sequences. BioTechniques. (2000) 28:11024–4. doi: 10.2144/00286ir01 10868275

[38] Ho TT Trinh VT Tran HX Le PTT Nguyen TT Hoang HTT . The immunogenicity of plant-based COE-GCN4pII protein in pigs against the highly virulent porcine epidemic diarrhea virus strain from genotype 2. Front Vet Sci. (2022) 9:940395. doi: 10.3389/fvets.2022.940395, 35967993 PMC9366249

[39] Liu X Lin CM Annamalai T Gao X Lu Z Esseili MA . Determination of the infectious titer and virulence of an original US porcine epidemic diarrhea virus PC22A strain. Vet Res. (2015) 46:109. doi: 10.1186/s13567-015-0249-1, 26408019 PMC4582625

[40] Liu X Zhang L Zhang Q Zhou P Fang Y Zhao D . Evaluation and comparison of immunogenicity and cross-protective efficacy of two inactivated cell culture-derived GIIa- and GIIb-genotype porcine epidemic diarrhea virus vaccines in suckling piglets. Vet Microbiol. (2019) 230:278–82. doi: 10.1016/j.vetmic.2019.02.018, 30827401

[41] Gutiérrez AH Martin WD Bailey-Kellogg C Terry F Moise L De Groot AS. Development and validation of an epitope prediction tool for swine (PigMatrix) based on the pocket profile method. BMC Bioinformatics. (2015) 16:290. doi: 10.1186/s12859-015-0724-8, 26370412 PMC4570239

[42] Hammer SE Ho CS Ando A Rogel-Gaillard C Charles M Tector M . Importance of the major histocompatibility complex (swine leukocyte antigen) in swine health and biomedical research. Annu Rev Anim Biosci. (2020) 8:171–98. doi: 10.1146/annurev-animal-020518-115014, 31846353

