# Supplementary material for: Design of multi-epitope vaccine based on G2c genotype PEDV antigen and its immunogenicity and protective effect in piglets
Source: Front Vet Sci. 2026 Oct 8;13:1942409. doi: 10.3389/fvets.2026.1942409 (PMC13688879; doi:10.3389/fvets.2026.1942409)
Supplement: Supplementary file 1 [file Supplementary_file_1.docx]

Supplementary Material

# 1 Protein sequence

MEAAAKGIINTLQKYYCRVRGGRCAVLSCLPKEEQIGKCSTRGRKCCRRKKEAAAKAVDGGDTAV

HEYGAEALERAGSIGYVPSQSGQVKIAGPGPGTTTIVYGRVGRSVNAGPGPGEGVFWVAKEGAKTE

PGPGPGQIRWRMRRGERIEQPGPGPGRDDLVAAVKDALKSKKGYPEFGSGKKTNFSMSIRKKRLES

VEVNSKKYKVATGVQVSQLKKGRVGRSVNASSGTGKKSFQDRGRKRVPLKKADLRYRTRKKGGFKN

FGDAEFVEKGVDARVRRHHHHHH..

## 1.1 Supplementary Tables

**Supplementary Table S1. Information table of 50 virus strains**

| **strain name** | **Place of origin** | **date** | **serial number** |
| --- | --- | --- | --- |
| CH/HNLYL/2021 | China | 2021 | ON960076.1 |
| CV777 | Belgium | 2001 | AF353511 |
| Attenuated DR13 | Korea | 2012 | JQ023162.1 |
| Virulent DR13 | Korea | 2012 | JQ023161.1 |
| 83P-5 | Japan | 2013 | AB548620.1 |
| KPEDV-9 | Korea | 2014 | KF898124.1 |
| SD-M | Shandong/China | 2012 | JX560761.1 |
| P5-V | Japan | 2018 | KY619780 |
| LW/L | China | 2010 | MK392335.1 |
| AJ1102 | Hubei/China | 2012 | JX188454.1 |
| GD-B | Guangdong/China | 2012 | JX088695.1 |
| XJ-DB2 | Xinjiang/China | 2014 | KM287429.1 |
| CV777 | Heilongjiang/China | 2012 | JN599150.1 |
| JS2008 | Jiangsu/China | 2008 | KC109141 |
| CH-S | Shanghai/China | 2011 | JN547228 |
| LZC | Gansu/China | 2007 | EF185992 |
| EAS1 | Thailand | 2014 | KR610991 |
| L00721-GER-2014 | Germany | 2014 | LM645057 |
| GER-L00719-2014 | Germany | 2014 | LM645058 |
| 15V010/BEL/2015 | Belgium | 2015 | KR003452.1 |
| FR-001-2014 | France | 2014 | KR011756 |
| NL/GD001/2015 | Netherlands | 2018 | MF974246.1 |
| PEDV_GER_L00862_2014 | Germany | 2018 | LT898447. |
| JS-2004-2 | Jiangsu/China | 2004 | AY653204 |
| GDjm17-1 | Guangdong/China | 2020 | MN368701.1 |
| KUIDL-PED-2014-007 | Korea | 2014 | KJ588062.1 |
| ON-007 | Canada | 2014 | KM189366 |
| PC21A | USA | 2015 | KR078299 |
| USA/Colorado/2013 | USA | 2013 | KF272920.1 |
| TW-Pingtung-63 | Taiwan/China | 2014 | KP276250.1 |
| AH2012 | Jiangsu/China | 2012 | KC210145 |
| CH-ZMDZY-11 | Henan/China | 2011 | KC196276 |
| YN144 | Hubei/China | 2015 | KT021232 |
| FL2013 | Guangdong/China | 2013 | KP765609 |
| CH/HNXJ/2016 | Gansu/China | 2017 | MF152596 |
| DT1 | Vietnam | 2017 | MG373532 |
| 13JM-291 | Japan | 2013 | KY619768 |
| YnP2 | Yunnan/China | 2017 | MG334003 |
| CH-hubei-2016 | Hubei/China | 2016 | KY928065 |
| FJqz16 | Guangdong/China | 2019 | MN368683.1 |
| GDsg16-1 | Guangdong/China | 2019 | MN368690.1 |
| YnP1 | Yunnan/China | 2016 | MG334002.1 |
| CH/JLDH/2016 | Jilin/China | 2016 | MF346935 |
| CH/SCXC/2018 | Sichuan/China | 2019 | MK598821.1 |
| SH-2015-921 | China | 2015 | KU710249 |
| CH/SCAZ10/2017 | Sichuan/China | 2017 | MH061339 |
| CH-HeN22-2022 | China | 2022 | OQ718902.1 |
| CH-HeN25-2023 | China | 2023 | OQ718905.1 |
| PEDV/CH/HN/NK1/2021 | China | 2022 | OP019340.1 |
| JX-SCAU2020 | China | 2021 | MW646416.1 |

**Supplementary Table S2. In silico physicochemical and immunological characterization of the MEV construct.**

| **Category** | **Parameter** | **Value** |
| --- | --- | --- |
| General characteristics | Sequence length | 285 aa |
|  | Molecular weight | 30.92 kDa |
|  | Theoretical pI | 10.36 |
| Stability & solubility | Instability index | 28.96 (Stable) |
|  | Aliphatic index | 58.84 |
|  | Grand average of hydropathy (GRAVY) | −0.830 (Hydrophilic) |
|  | SOLpro solubility probability | 0.770 (Soluble) |
| Antigenicity & allergenicity | ANTIGENpro score | 0.717 (Antigenic) |
|  | VaxiJen v2.0 score | 0.686 (Antigenic) |
|  | AllerTOP v2.0 prediction | Non-allergen |
| Secondary structure | α-helix | 23.16% |
|  | Extended strand | 24.21% |
|  | β-turn | 11.23% |
|  | Random coil | 41.40% |

**Supplementary Table S3. Predicted conformational B-cell epitopes in the MEV tertiary structure.**

| **No.** | **Residues** | **Number of residues** | **score** |
| --- | --- | --- | --- |
| A | A:H281,A:H284,A:H285 | 3 | 0.779 |
| B | A:E198,A:V199,A:N200,A:S201,A:K202,A:K203,A:Y204,A:K205,  A:L215,A:K217,A:G218,A:V220,A:G221,A:R222,A:S223,A:V224,  A:N225,A:A226,A:S227,A:S228,A:G229,A:T230,A:G231,A:K232,  A:K233,A:S234,A:F235,A:Q236,A:D237,A:R238,A:G239,A:R240,  A:K241,A:R242,A:V243,A:P244,A:L245,A:K246,A:K247,A:A248,  A:D249,A:L250,A:R251,A:Y252,A:R253,A:T254,A:R255,A:K256,  A:K257,A:G258,A:G259,A:F260,A:K261,A:N262,A:F263,A:G264,  A:D265,A:A266,A:E267,A:K271 | 60 | 0.755 |
| C | A:Y15,A:V18,A:R19,A:G20,A:G21,A:R22,A:A24,A:V25,A:S27,  A:C28,A:L29,A:P30,A:K31,A:E32,A:E33,A:Q34,A:I35,A:G36,  A:K37,A:C38,A:S39,A:T40,A:R41,A:G42,A:R43,A:K44,A:C45 | 27 | 0.722 |
| D | A:V105,A:R107,A:S108,A:V109,A:N110,A:A111,A:G112,A:P113,  A:G114,A:E117,A:W121,A:K124,A:G126,A:A127,A:K128,A:T129,  A:E130,A:P131,A:G132,A:P133,A:G134,A:P135,A:G136,A:Q137,  A:I138,A:R139,A:W140,A:R141,A:M142,A:R143,A:R144,A:G145,  A:E146,A:R147,A:I148,A:E149,A:Q150,A:P151,A:G152 | 39 | 0.716 |
| E | A:K182,A:T183,A:N184,A:M187,A:S188,A:K191 | 6 | 0.634 |
| F | A:E1,A:A4,A:K5 | 3 | 0.628 |

## 1.2 Supplementary Figures

**Supplementary Figure S1. Schematic representation of the methodology and tools used in this study**

B

**Supplementary Figure S2. Secondary-structure prediction of MEV using PSIPRED**

**Supplementary Figure S3.Tertiary-structure analysis of MEV**

**Supplementary Figure S4. Conformational B-cell epitope mapping on the MEV tertiary model**
